# Draft Genome Sequence of Shewanella sp. ECSMB14101, Isolated from the East China Sea

**DOI:** 10.1128/genomeA.01388-14

**Published:** 2015-01-15

**Authors:** Jin-Long Yang, Xing-Pan Guo, De-Wen Ding

**Affiliations:** a Key Laboratory of Exploration and Utilization of Aquatic Genetic Resources, Shanghai Ocean University, Ministry of Education, Shanghai, China; b Marine Ecology Research Center, the First Institute of Oceanography, State Oceanic Administration, Qingdao, China

## Abstract

Shewanella sp. ECSMB14101 was isolated from marine biofilms formed on the East China Sea. The draft genome sequence comprises 4,272,451 bp with a G+C content of 49.82%. Information on this draft genome will contribute to the understanding of bacterium-animal interactions.

## GENOME ANNOUNCEMENT

Members of the genus Shewanella compose a diverse group of facultative anaerobic bacteria widely distributed in marine and freshwater environments (1). Given their widespread distribution in environments where organic matter is actively degraded, members of the genus Shewanella may be viewed as useful model organisms for obtaining systems-level insight into the carbon-cycling process (2). In addition, Shewanella sp. ECSMB14101 is just one of a host of biofouling microorganisms that can be found in marine biofilms, and can induce the settlement of larvae (3) and plantigrades (4) of the mussel Mytilus coruscus, an important fouling and aquaculture species in the East China Sea (5). More than 40 Shewanella genomes have been fully sequenced to data, and 25 strains have complete whole-genome sequences. In order to understand the interaction between the role of Shewanella sp. ECSMB14101 and the settlement mechanism of the mussel Mytilus coruscus, the draft genome of Shewanella sp. ECSMB14101 was sequenced.

The strain of Shewanella sp. ECSMB14101 was isolated from marine biofilms formed on the East China Sea (122°46′E; 30°43′N, water depth of 0.5 m), and the 16S rRNA sequences of Shewanella sp. ECSMB14101 shared 100% similarity with Shewanella marisflavi (accession no. AY485224) (3). Here, we present the draft genome sequence of the strain ECSMB14101, generated using the Illumina MiSeq platform by Shanghai Majorbio Pharm Technology Co., Ltd. (Shanghai, China) with a paired-end library. After trimmed and merged, the reads were *de novo* assembled with GS De Novo Assembler v2.8. Open reading frames (ORFs) were predicted using the program Glimmer 3.02 (6). All ORFs were then annotated by comparison with NCBI-NR and KEGG using BLASTp (BLAST 2/2/28+). The tRNA and rRNA were predicted by the program tRNAscan-SE v1.3.1 and Barrnap 0.4.2 (http://www.vicbioinformatics.com/software.barrnap.shtml), respectively (7).

The draft genome sequence of the strain ECSMB14101 consists of 81 contigs (>200 bp) of 4,272,451 bp, and the largest contig assembled is 587,120 bp. The *N*_50_ and *N*_90_ quality measurement of the contigs were 159,739 bp and 43,702 bp, respectively. The draft genome sequence has a G+C content of 49.82%. The genome contains 3,647 predicted protein-coding sequences, 90 tRNA genes for 20 amino acids, and 4 rRNA genes. The genome sequence of Shewanella sp. ECSMB14101 will provide a starting point for understanding the relationship between the settlement-associated contractile structure genes and the settlement process of the mussel M. coruscus.

### Nucleotide sequence accession numbers.

This whole-genome shotgun project has been deposited at DDBJ/EMBL/GenBank under the accession no. JSFF00000000. The version described in this paper is the first version, JSFF01000000.
